# Self-compassion, adverse childhood experiences and perceived stress on college students’ mental health: The role of resilience

**DOI:** 10.1371/journal.pone.0353462

**Published:** 2026-07-13

**Authors:** Hainan Li, Rong Kong, Lifang Wang

**Affiliations:** 1 Department of Psychology, Tianjin Normal University, Tianjin, China; 2 School of Humanity and Law, University of Emergency Management, Langfang, Hebei, China; 3 Center of Psychological Education and Counselling, Taiyuan Institute of Technology, Taiyuan, Shanxi, China; 4 Student Affairs Office, University of Emergency Management, Langfang, Hebei, China; Tung Wah College, HONG KONG

## Abstract

**Background:**

The college environment presents various academic and life pressures that affect students’ mental health. Previous studies have examined self-compassion, perceived stress, and adverse childhood experiences separately, but few have integrated them within a unified resilience framework. Drawing on the stress-buffering theory, this study aims to examine the relationships among self-compassion, ACEs, perceived stress, and mental health, and to investigate the mediating role of resilience. The study develops an integrative framework that simultaneously considers risk and protective psychological mechanisms, thereby extending previous research by examining the mediating role of resilience in the associations of early adversity, stress perception, and self-compassion with mental health among university students.

**Methods:**

This cross-sectional study employed an online self-report survey conducted via the Questionnaire Star platform (www.Sojump.com). Using a convenience sampling method, college students from two universities in China completed the Self-Compassion Scale, Adverse Childhood Experiences Scale, Perceived Stress Scale, Resilience Scale, and Mental Health Scale. A total of 657 valid questionnaires were obtained. Correlations among variables were analyzed using SPSS Statistics 26, and mediation effects were tested with Model 4 of the PROCESS 4.0 macro.

**Results:**

Self-compassion was positively associated with mental health, while adverse childhood experiences and perceived stress were negatively associated with mental health. Resilience played a mediating role, linking these factors to mental health outcomes.

**Conclusion:**

This study underscores the importance of resilience in mental health and provides a theoretical basis for interventions in higher education, emphasizing resilience enhancement to support student well-being.

## 1. Introduction

Mental health (MH) issues have become a significant factor affecting college students’ academic performance, daily life, and future development [1]. According to the World Health Organization (WHO), psychological conditions frequently arise before individuals reach 24 years, highlighting the pivotal influence of the university period on mental development [2]. Research on MH challenges among Chinese college students has indicated that 21.4% of individuals in this demographic experience MH issues [3]. Deteriorated mental well-being strongly correlates with self-injurious actions and suicidal risk, as those struggling with severe psychological distress are more susceptible to engaging in self-harm and facing heightened suicide vulnerability [4]. Recent findings from the WHO World Mental Health International College Student (WMH-ICS) project showed high prevalence rates of mental disorders among university students from 19 universities across eight countries [5]. About 35% reported at least one lifetime disorder and 31% reported at least one 12-month disorder, highlighting the global relevance of student mental health concerns. Therefore, exploring effective strategies to improve college students’ MH has become critical.

Currently, research on the factors influencing college students’ MH mainly focuses on internal individual factors and external environmental factors. Internal individual factors such as perceived stress (PS), self-compassion (SC), resilience, and self-esteem [6–9] are considered pivotal when students face challenges such as academic pressure, emotional distress, and identity issues. PS, as the individual’s subjective assessment of environmental stress, is associated with MH [10]. SC helps individuals reduce negative emotions and enhance psychological resilience when faced with difficulties [6,11]. External environmental factors, such as adverse childhood experiences (ACEs), parenting styles, and interpersonal relationships [12–14], shape an individual’s psychological resources and further affect their MH. Specifically, ACEs have been found to be closely related to emotional disorders and stress perception among college students [15].

Resilience, as a key psychological resource, enables individuals to adapt positively to adversity and maintain mental well-being [16]. Although previous studies have separately examined the associations between SC, ACEs, PS, and mental health [17–21], few have investigated how these factors operate together within one framework. Integrating these variables is essential because SC functions as an internal protective factor, whereas ACEs and PS represent early and current stressors that may weaken resilience. Guided by the Transactional Model of Stress and Coping [22], which emphasizes that stress outcomes are associated with both environmental demands and individual coping resources, this study examines a framework in which resilience mediates the associations of SC, ACEs, and PS with college students’ mental health. By integrating these risk and protective factors within a single framework, the present study extends previous research and provides a more comprehensive understanding of how these factors are associated with psychological well-being among college students.

### 1.1 SC and MH

Neff [23] defines SC as treating oneself with kindness in the face of difficulties and failure, being able to accept one’s flaws and shortcomings, evaluating oneself objectively, and showing kindness and care during times of sadness and frustration. Individuals with higher levels of SC are typically able to view negative life events from a more balanced and objective perspective, and as a result, they experience fewer intense negative emotions [24]. Numerous studies have demonstrated a positive association between SC and MH [25–27]. According to the stress-buffering theory [28], SC acts as an internal coping resource that regulates emotions and mitigates the negative effects of stress, fostering emotional resilience and psychological well-being. For instance, Kotera and Ting [26] found that among Malaysian university students, SC was the strongest independent predictor of MH, suggesting that SC training can enhance students’ well-being. Similarly, Min, Jianchao and Mengyuan [25] reported that SC and informal help-seeking behaviors were significantly associated with MH among Chinese postgraduate students. Given the increasing competition among college students and the escalation of psychological symptoms, fostering SC and the ability to seek help are critically important. In conclusion, SC, as an important psychological resource, not only facilitates individual growth and development in the face of challenges but also helps individuals effectively improve their MH.

### 1.2 ACEs and MH

Before the age of 18, individuals may experience ACEs, which include traumatic events such as abuse, neglect, and family dysfunction [29]. These stressors disrupt development by affecting the neuroendocrine-immune system, increasing physiological burden and harming MH [30]. According to the stress-buffering theory, individuals with fewer protective resources are more vulnerable to stress [31]. ACEs weaken coping and emotional regulation, heightening psychological distress [32]. However, resilience can buffer these effects by fostering adaptive coping strategies [33]. A large body of research has confirmed the significant negative impact of ACEs on college students’ MH [15,34–36]. For instance, Bhargav and Swords [15]examined European and non-European university students and found that ACEs were common and strongly associated with poorer MH outcomes, including psychological distress, suicidal ideation, and reduced resilience. Small, Kim and Yu [35] studied the MH risk factors of college students in Sierra Leone and found that ACEs were significantly associated with anxiety, depression, and other MH issues. Huang, Tan, Cheung and Hu [34] explored the impact of ACEs on Chinese college students’ MH, concluding that students with ACEs were more likely to develop MH problems, with ACEs significantly negatively affecting their well-being. At the same time, ACEs are not a homogeneous construct, as different types or patterns of childhood adversity may have distinct associations with later psychological adaptation [37]. Thus, although the cumulative ACE score is widely used and analytically practical, it represents a simplified operationalization of a complex developmental risk construct. In conclusion, ACEs are associated with lower MH in college students. Understanding how ACEs are linked to MH in college students is key to detecting initial risk elements associated with MH challenges.

### 1.3 PS and MH

PS refers to an individual’s personal assessment of the stress levels within their living environment [38], reflecting their subjective response to stressors [39]. Among college students, the prevalence of PS is notable, given the numerous difficulties they navigate, such as educational pressures, societal expectations, and economic hardships [40]. Excessive PS has been consistently linked to a range of MH problems, including depression, anxiety, and suicidal behaviors [7,10,41]. For example, Kaya [42] examined Turkish college students and found that higher PS were significantly associated with depression severity, indicating that stress perception plays a major role in students’ psychological distress. Similarly, He, Tu, Zhao and He [10] studied Chinese college students and reported that elevated PS weakened self-control and negatively affected MH. Overall, PS is negatively associated with students’ MH. Clarifying its mechanisms can help explain how stress contributes to psychological distress.

### 1.4 The mediating role of resilience

Resilience refers to the capacity to navigate adversity, allowing individuals to bounce back from challenges like stress, anxiety, and depression [43]. Individuals with high resilience are more adaptable and capable of managing challenges effectively, which helps reduce the risk of MH problems [44]. Research findings indicate that resilience and MH are deeply interconnected [18,45–49]. For example, Ma, Liu, Raymond Sum, Gao, Li, Choi, et al. [18] found that college students with stronger resilience showed greater confidence and adaptability in difficult circumstances, leading to better MH. Furthermore, resilience is seen as an important defense mechanism that helps individuals resist emotional distress after experiencing setbacks, playing a key role in promoting MH [46]. At the same time, emerging research suggests that resilience may not be a purely unitary construct, but may instead involve multiple dimensions or sources of adaptive capacity [50]. This perspective underscores the complexity of resilience and provides a broader context for understanding its association with mental health.

Resilience is related to both internal and external factors. Among internal factors, SC is an important psychological resource that enhances resilience and emotional well-being [24,51–53]. Hou, Qu, Bu, Chen, Liu and Yu [24] pointed out that SC significantly improves students’ emotional states and MH by providing psychological resources to cope with stressors. They further indicated that SC reduces psychological distress and enhances resilience and well-being [53]. Furthermore, SC promotes emotional regulation and adaptive coping strategies, reinforcing its buffering effect against stress [24]. Existing studies have confirmed the mediating role of resilience in the relationship between SC and MH [21,24,52,53]. For example, Harmanci and Akdeniz [21] studied Turkish healthcare workers and found that resilience mediated the effect of SC on MH, indicating that SC alleviates stress partly through strengthening resilience.

External factors, such as adverse ACEs and PS, tend to reduce resilience and impair MH. According to the stress-buffering theory, resilience serves as a key psychological resource that mitigates the adverse impact of both early and current stressors. Individuals exposed to higher levels of ACEs or PS are more likely to experience emotional distress when their resilience is insufficient to buffer these pressures [20]. Rather than directly impairing MH, ACEs may reduce resilience by shaping maladaptive coping patterns developed in early life [33,54–56]. For instance, Okwori [56] found that lower resilience among individuals with ACEs was associated with greater vulnerability to psychological distress. Similarly, PS has been linked to diminished resilience and increased emotional exhaustion [20,49,57,58]. Loreto Lara-Cabrera, Betancort, Amparo Munoz-Rubilar, Rodriguez Novo and De Las Cuevas [58] reported among Spanish nurses that resilience mediated the association between PS and MH. Overall, resilience functions as a central adaptive mechanism linking protective (SC) and risk (ACEs, PS) factors to MH.

### 1.5 Current study

**Fig 1:**
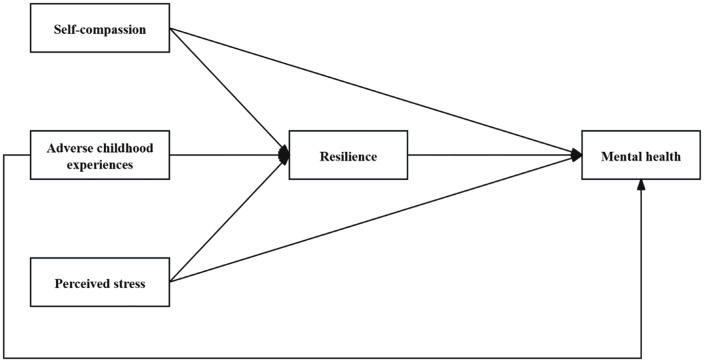
Hypothetical model.

Based on the stress-buffering theory [28], this study examines the mediating role of resilience in the relationships among SC, ACEs, PS, and MH in college students. SC represents an internal protective factor that may enhance resilience and psychological well-being, whereas ACEs and PS represent external and current stressors that may weaken resilience and increase psychological distress. Resilience functions as a buffer that reduces the negative association between ACEs and PS with MH, while strengthening the positive association between SC and MH. Based on these theoretical assumptions and previous empirical findings, the following hypotheses were formulated (see Fig 1):

H1: SC is positively associated with college students’ MH.

H2: ACEs are negatively associated with college students’ MH.

H3: PS is negatively associated with college students’ MH.

H4: Resilience mediates the relationship between SC and college students’ MH.

H5: Resilience mediates the relationship between ACEs and college students’ MH.

H6: Resilience mediates the relationship between PS and college students’ MH.

## 2. Methods

### 2.1 Sample and data collection

To enhance sample diversity and improve the applicability of the findings, this study employed a convenience sampling strategy for questionnaire distribution and collection. The sampling frame was based on official class rosters obtained from two certain universities in North China, covering students from humanities, social sciences, natural sciences, and management majors. Participants were recruited from available classes across different academic years (freshman, sophomore, junior, and senior) and disciplines through class-based access and voluntary participation. Between October and November 2024, data were obtained through the Questionnaire Star platform (www.Sojump.com), an online survey tool, to assess the hypothesized model. This two-month period was chosen to ensure sufficient participant accessibility and time to meet the predetermined sample size within a stable academic context. October to November represents a regular teaching period in Chinese universities, during which students’ academic schedules and psychological states remain relatively stable, thus reducing potential contextual variability. All data were collected before any statistical analyses were conducted, and no interim analyses were performed during the data collection phase. This study was approved by North China Institute of Science and Technology (Approval Number:2024–0015). Prior to administering the survey, all participants were informed of the study purpose, the voluntary nature of participation, and the confidentiality of their data. The participants received oral and written information and provided written informed consent before participating in the study. For logistical purposes, the research team coordinated with class instructors to distribute the survey link to eligible students. Instructors assisted only with communication and questionnaire distribution and were not involved in participant screening or response monitoring. Because participation was voluntary and anonymous, students could freely decide whether to complete the questionnaire, and instructors were unable to identify who responded.

Inclusion criteria were as follows: (1) full-time undergraduate students; (2) enrolled in a degree program at a comprehensive university in China; and (3) voluntary participation with informed consent. Exclusion criteria included: (1) a self-reported history of diagnosed mental disorder, and (2) duplicate or invalid responses detected during data screening. The exclusion of students with a self-reported diagnosed mental disorder was intended to reduce potential confounding associated with clinically diagnosed psychiatric conditions; however, it also means that the sample was restricted to a non-clinical college student population. According to the sample size calculation method proposed by Kline [59], at least 10 respondents should answer each survey item. This study’s questionnaire included 52 items, and considering an estimated sample dropout rate of 20%, the required sample size was calculated to be 624 participants (52 items × 10 respondents + 20% × 52 items × 10 respondents). To further ensure that the final number of valid responses met the required threshold, 700 questionnaires were distributed, slightly exceeding the calculated minimum to account for possible non-response and invalid submissions. Out of 700 distributed questionnaires, 673 were collected. Prior to analysis, data were screened for completeness and quality. Responses with more than 20% missing values were excluded, and no data imputation procedures were applied, as the proportion of missing data in the retained sample was minimal. After data cleaning, 16 responses were removed due to more than 20% missing data or extreme response patterns (e.g., choosing “Strongly Agree” or “Strongly Disagree” on over 80% of items). The final valid sample included 657 participants. All data were collected anonymously via a secure online survey platform, and no personally identifiable information was recorded. Detailed demographic characteristics of the participants are summarized in Table 1.

**Table 1:** Demographic characteristics of the sample.

Category	Subcategory	Number	Percentage
Gender	Male	199	30.3%
	Female	458	69.7%
Age	< 18	12	1.8%
	18-20	481	73.2%
	20-22	132	20.1%
	> 22	32	4.9%
Grade	Freshman	366	55.7%
	Sophomore	144	21.9%
	Junior	104	15.8%
	Senior	43	6.5%
Only Child	Yes	204	31.1%
	No	453	68.9%
Parents’ Education Level	Primary School	101	15.4%
	Junior High	266	40.5%
	High School	153	23.3%
	University	137	20.9%

### 2.2 Measurement tools

The questionnaire was structured into two sections: the initial segment captured participants’ demographic data, while the subsequent section recorded self-reported information on multiple psychological constructs. Validated scales were employed to measure these constructs, with necessary refinements made in alignment with the study’s scope and objectives. In addition to demographic variables, the questionnaire assessed key constructs, including SC, ACEs, PS, resilience, and MH.

The Self-Compassion Scale (SCS) was modified following the framework proposed by Wang, Tang, Lv, Tao, Liu, Zhang, et al. [60]. This scale captures cognitive, affective, and behavioral patterns linked to SC, categorized into six facets: Over-identification, Self-Kindness, Mindfulness, Isolation, Common Humanity, and Self-Judgment. It consists of 12 items (e.g., “When I fail at something important to me, I get consumed by feelings of inadequacy”). Items 1, 4, 8, 9, and 12 require reverse scoring. Responses are measured using a 5-point Likert scale from 1 (Never) to 5 (Always), where elevated scores reflect stronger SC. The scale exhibited satisfactory psychometric properties, with a Cronbach’s alpha of 0.781 and a KMO value of 0.842. To further assess the construct validity of the scale, confirmatory factor analysis (CFA) was conducted, and the model demonstrated a good fit to the data (χ²/df = 3.025, RMSEA = 0.056, GFI = 0.993, AGFI = 0.940, IFI = 0.992, CFI = 0.992, TLI = 0.939).

The Adverse Childhood Experiences scale was adapted based on the study by Huang, Yang, Geng, Chen, Cheung, Deng, et al. [61]. It primarily assesses ACEs up to the first 18 years of life, with three dimensions: Abuse (3 items), Neglect (2 items), and Family Challenges (5 items), totaling 10 items (e.g., “During your childhood, were you emotionally abused?”). The scale uses a binary scoring system, where each item is answered as “Yes” or “No”. Each “Yes” response contributes 1 point, whereas a “No” response carries 0 points. The cumulative score spans from 0 to 10, where higher values denote a greater exposure to ACEs. In the present study, ACEs were operationalized using the cumulative total score, which is a widely used and defensible approach for capturing overall childhood adversity, although it may simplify the heterogeneity of different adverse experiences. The Cronbach’s alpha coefficient was 0.700, and the KMO value stood at 0.750, demonstrating satisfactory reliability and validity. A CFA indicated a good model fit (χ²/df = 2.257, RMSEA = 0.044, GFI = 0.988, AGFI = 0.962, IFI = 0.980, CFI = 0.980, TLI = 0.947).

The Perceived Stress Scale (PSS-10) was adapted based on the study by Yang, Liu, Shi, Dong, Cheng and Li [62]. It assesses the frequency with which participants have experienced unpredictable or overwhelming events in their lives during the past month, consisting of 10 items (e.g., “In the past month, have you felt upset because of something unexpected?”). Reverse scoring applies to items 4, 5, 7, and 8. This scale employs a 5-point Likert system, with responses ranging from 1 (Never) to 5 (Always). The overall score extends from 10 to 50, where higher values signify increased PS levels. With a Cronbach’s alpha of 0.857 and a KMO value of 0.944, the results reflect strong reliability and validity. Model fit indices also supported satisfactory construct validity (χ²/df = 1.825, RMSEA = 0.035, GFI = 0.991, AGFI = 0.971, IFI = 0.995, CFI = 0.995, TLI = 0.987).

The Resilience Scale (CD-RISC) was adapted from Su and He [63] and measures college students’ ability to cope with adversity. This instrument comprises 10 statements (e.g., “I adjust well to new situations”), each rated on a scale from 1 to 5. The total score, ranging from 10 to 50, serves as an indicator of resilience, where higher values denote greater adaptability and psychological strength. In this study, resilience was treated as a total-score construct to reflect individuals’ overall adaptive capacity. Although this approach is appropriate for the present analytic purpose, it should be noted that resilience may also involve multiple dimensions that are not fully captured by a single composite score. The scale demonstrated excellent reliability and validity, with a Cronbach’s alpha of 0.928 and a KMO value of 0.883. The CFA yielded acceptable fit indices (χ²/df = 2.475, RMSEA = 0.047, GFI = 0.980, AGFI = 0.960, IFI = 0.990, CFI = 0.990, TLI = 0.983).

The Mental Health Scale was adapted based on the study by Liu, Sun, Guo and Zheng [64], as depression assessment is regarded as one of the most important indicators of MH [65,66]. This study used the Chinese version of the World Health Organization Five Happiness Index (WHO-5) to assess depressive symptoms and reflect participants’ MH. The scale includes 5 items (e.g., “In the past two weeks, I have felt cheerful and relaxed”), and a 7-point Likert scale is used, ranging from 1 (Never) to 7 (Always). A higher total score indicates better MH. Cronbach’s alpha was 0.934, and the KMO value was 0.883, indicating excellent reliability and validity. Structural validity testing showed that the model fit the data well (χ²/df = 2.034, RMSEA = 0.040, GFI = 0.996, AGFI = 0.982, IFI = 0.999, CFI = 0.999, TLI = 0.996).

The translation process for the scales was carried out by two experts: one was an expert in the content area with proficiency in both English and Chinese, and the other was a linguist specializing in the Chinese language. These experts translated the English versions of the scales into Chinese from both a content and linguistic perspective. During the translation process, the two versions were reviewed and discussed to coordinate any significant differences, and a unified final version was created. A pilot test was conducted with 20 Chinese college students to refine the translation. The process allowed for resolving any issues, resulting in the completion of the final Chinese version of the scales.

### 2.3 Data analysis

Statistical analysis was performed using SPSS Statistics 26 software to conduct descriptive statistics, correlation analysis, reliability and validity tests, and other analyses. To assess the measurement model, CFA was conducted to identify the observed variables for each latent variable and evaluate the structural, convergent, and discriminant validity. Prior to the multivariable analyses, common method bias and multicollinearity were examined to ensure data quality. Harman’s single-factor test revealed that the first factor accounted for 29.68% of the total variance, which is below the 40% threshold [67], suggesting that common method bias was not a concern. Multicollinearity was assessed using variance inflation factor (VIF) values, which ranged from 1.129 to 2.164, all well below the recommended cutoff of 3.3 [68], indicating no multicollinearity issues. Meanwhile, to investigate how resilience mediates the associations of SC, ACEs, and PS on college students’ MH, mediation analysis was carried out using Model 4 in the PROCESS 4.0 plugin. The Bootstrap method with 5,000 resamples used to estimate 95% confidence intervals (95% CI) for the indirect effects, thereby enhancing the robustness of the mediation analysis.

## 3. Results

### 3.1 Differences across demographic variables

Table 2 presents group differences in SC, ACEs, PS, resilience, and MH across demographic variables. The sample included students with diverse demographic backgrounds in terms of gender, age, grade, family structure, and parental education level. Independent-samples *t*-tests and one-way ANOVAs were then conducted to examine variations in SC, ACEs, PS, resilience, and MH across demographic subgroups. Results showed that female students reported lower PS than males (*p* = 0.031). Significant age differences were found in SC, PS, resilience, and MH (*p* < 0.05), with older students scoring higher on SC, resilience, and MH, and lower on PS. Seniors also exhibited higher SC and lower PS than freshmen and sophomores (*p* < 0.01). Only children reported lower PS and higher resilience and MH than non–only children (*p* < 0.05). Furthermore, students whose parents had higher educational levels scored higher on SC, resilience, and MH, and lower on PS (*p* < 0.05). Overall, these findings indicate that several key study variables differed across demographic characteristics, suggesting that demographic characteristics may be associated with levels of self-compassion, perceived stress, resilience, and mental health among college students. Although several demographic differences reached statistical significance, the corresponding effect sizes were generally small (Cohen’s *d*s = 0.11–0.24; η^2^s = 0.013–0.032), indicating that the magnitudes of these differences were modest. Therefore, demographic variables were included as covariates in subsequent analyses.

**Table 2:** Group differences in SC, ACEs, PS, resilience, and MH.

Category	Subcategory	Number	SC	ACEs	PS	Resilience	MH
Gender	Male	199	3.39 ± 0.53	1.92 ± 0.15	2.64 ± 0.63	3.77 ± 0.80	4.60 ± 1.33
	Female	458	3.41 ± 0.48	1.92 ± 0.13	2.53 ± 0.58	3.68 ± 0.65	4.74 ± 1.15
	*t*		−0.540	0.009	2.168	1.567	−1.327
	*p*		0.590	0.993	0.031	0.118	0.185
	Cohen’s *d*		−0.046	0.001	0.184	0.133	−0.113
Age	1. < 18	12	3.13 ± 0.58	1.93 ± 0.10	2.59 ± 0.51	3.66 ± 0.98	4.53 ± 1.67
	2. 18-20	481	3.38 ± 0.48	1.91 ± 0.14	2.60 ± 0.59	3.66 ± 0.69	4.62 ± 1.21
	3. 20-22	132	3.49 ± 0.51	1.93 ± 0.14	2.51 ± 0.57	3.82 ± 0.69	4.86 ± 1.17
	4. > 22	32	3.64 ± 0.54	1.96 ± 0.08	2.26 ± 0.67	3.99 ± 0.75	5.16 ± 1.11
	*F*		5.676	1.210	3.584	3.704	3.052
	*p*		<0.001	0.305	0.014	0.012	0.028
	*LSD*		1 < 3*, 4** 2 < 3*, 4**		2 < 4** 3 < 4*	2 < 3*, 4**	2 < 3*, 4*
	η²		0.025	0.006	0.016	0.017	0.014
Grade	1. Freshman	366	3.38 ± 0.48	1.92 ± 0.13	2.55 ± 0.56	3.68 ± 0.68	4.66 ± 1.20
	2. Sophomore	144	3.34 ± 0.44	1.90 ± 0.15	2.71 ± 0.60	3.63 ± 0.71	4.60 ± 1.22
	3. Junior	104	3.50 ± 0.58	1.91 ± 0.16	2.44 ± 0.64	3.84 ± 0.74	4.90 ± 1.26
	4. Senior	43	3.68 ± 0.48	1.96 ± 0.10	2.44 ± 0.61	3.85 ± 0.69	4.84 ± 1.10
	*F*		7.244	1.718	5.447	2.578	1.632
	*p*		<0.001	0.162	0.001	0.053	0.181
	*LSD*		1 < 3*, 4*** 2 < 3**, 4*** 3 < 4*	2 < 4*	1 < 2** 3 < 2*** 4 < 2**	1 < 3* 2 < 3*	
	η²		0.032	0.008	0.024	0.012	0.007
Only Child	Yes	204	3.46 ± 0.50	1.93 ± 0.13	2.49 ± 0.61	3.82 ± 0.68	4.89 ± 1.28
	No	453	3.39 ± 0.49	1.91 ± 0.14	2.60 ± 0.58	3.66 ± 0.70	4.61 ± 1.16
	*t*		1.644	1.735	−2.170	2.719	2.836
	*p*		0.101	0.083	0.030	0.007	0.005
	Cohen’s *d*		0.139	0.146	−0.183	0.229	0.239
Parents’ Education Level	1. Primary School	101	3.35 ± 0.47	1.89 ± 0.17	2.64 ± 0.54	3.68 ± 0.69	4.48 ± 1.28
	2. Junior High	266	3.36 ± 0.51	1.91 ± 0.15	2.62 ± 0.57	3.59 ± 0.69	4.59 ± 1.20
	3. High School	153	3.48 ± 0.49	1.94 ± 0.12	2.51 ± 0.61	3.79 ± 0.71	4.87 ± 1.12
	4. University	137	3.46 ± 0.48	1.94 ± 0.10	2.46 ± 0.63	3.85 ± 0.70	4.85 ± 1.25
	*F*		2.975	4.984	3.200	5.104	3.553
	*p*		0.031	0.002	0.023	0.002	0.014
	*LSD*		1 < 3*, 2 < 3*	1 < 3, 4* 2 < 3, 4*	4 < 1, 2*	2 < 3**, 4***	1 < 3, 4* 2 < 3,4*
	η²		0.013	0.022	0.014	0.023	0.016

Note: *p < 0.05, **p < 0.01, ***p < 0.001.

### 3.2 Descriptive statistics and correlation analysis

Based on the descriptive statistics and correlation analysis provided in Table 3, significant relationships were found between SC, ACEs, PS, resilience, and MH (*p* < 0.001, N = 657). Specifically, for SC, the mean score was 3.41 (SD = 0.49), showing a strong positive relationship with MH and resilience, and a notable negative relationship with PS and ACEs. PS had a mean score of 2.56 (SD = 0.59), which was positively associated with ACEs and negatively associated with both MH and resilience. The mean score for MH was 4.69 (SD = 1.21), with a positive association to resilience and a negative one with ACEs. Resilience, with a mean score of 3.71 (SD = 0.70), was negatively correlated with ACEs.

**Table 3:** Descriptive statistics and correlation coefficients for SC, ACEs, PS, resilience, and MH.

	M ± SD	SC	PS	MH	Resilience	ACEs
SC	3.41 ± 0.49	1				
PS	2.56 ± 0.59	−0.664^***^	1			
MH	4.69 ± 1.21	0.579^***^	−0.709^***^	1		
Resilience	3.71 ± 0.70	0.619^***^	−0.637^***^	0.638^***^	1	
ACEs	1.92 ± 0.14	−0.282^***^	0.327^***^	−0.303^***^	−0.242^***^	1

Note: N = 657, ***p < 0.001.

### 3.3 Confirmatory factor analysis

Table 4 displays the findings of the Confirmatory Factor Analysis (CFA). All indices in the final model fall within acceptable ranges. When taking all results into account, the measurement model’s overall fit matches the theoretical expectations. In general, the model demonstrates a satisfactory fit, and the constructed model is deemed acceptable.

**Table 4:** Model fit indices for the structural equation model examining the mediating role of resilience.

Fit index	Reference value	Final model
CMIN/DF	<5	4.091
RMSEA	0.05-0.08	0.069
GFI	>0.85	0.892
AGFI	>0.85	0.862
CFI	>0.85	0.912
IFI	>0.85	0.913
TLI	>0.85	0.908

Note: CMIN: chi-square value, DF: degrees of freedom, RMSEA: root mean square error of approximation, GFI: goodness-of-fit index, AGFI: adjusted goodness-of-fit index, NFI: normed fit index, CFI: comparative fit index, IFI: incremental fit index, TLI: Tucker-Lewis index.

### 3.4 Mediating effect analysis

**Fig 2:**
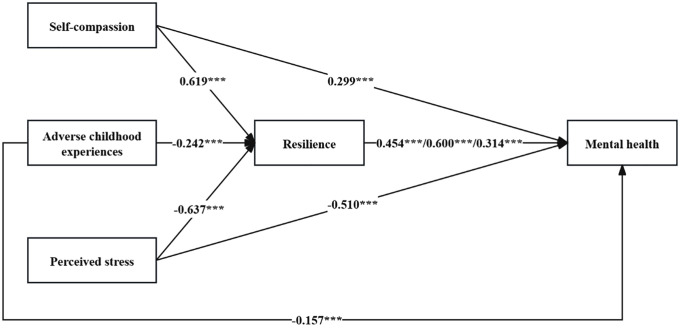
Results of the mediation model.

After adjusting for factors such as gender, age, and grade, the results of the regression analysis based on standardized data are shown in Table 5 and Fig 2. First, when SC was the independent variable, SC was significantly positively associated with resilience (*β* = 0.619, *p* < 0.001) and MH (*β* = 0.299, *p* < 0.001), and resilience was significantly positively associated with MH (*β* = 0.454, p < 0.001). Second, when ACEs were the independent variable, ACEs were significantly negatively associated with resilience (*β* = −0.242, *p* < 0.001) and MH (*β* = −0.157, *p* < 0.001), whereas resilience was significantly positively associated with MH (*β* = 0.600, *p* < 0.001). Additionally, when PS was the independent variable, PS was significantly negatively associated with resilience (*β* = −0.637, *p* < 0.001) and MH (*β* = −0.510, *p* < 0.001), and resilience was significantly positively associated with MH (*β* = 0.314, *p* < 0.001).

**Table 5:** Regression results for associations among SC, ACEs, PS, resilience, and MH.

Outcome Variable	Predict Variable	*β*	SE	*t*	Bootstrap 95% CI		R^2^	F
					LLCI	ULCI		
Resilience	SC	0.619	0.031	20.173***	0.559	0.679	0.619	406.952***
	ACEs	−0.242	0.038	−6.388***	−0.317	−0.168	0.242	40.809***
	PS	−0.637	0.030	−21.142***	−0.696	−0.578	0.637	446.978***
MH	SC	0.299	0.037	8.179***	0.227	0.370	0.680	281.331***
	Resilience	0.454	0.037	12.423***	0.382	0.525		
	ACEs	−0.157	0.030	−5.176***	−0.217	−0.098	0.656	247.487***
	Resilience	0.600	0.030	19.739***	0.541	0.660		
	PS	−0.510	0.034	−15.178***	−0.576	−0.444	0.749	419.273***
	Resilience	0.314	0.034	9.346***	0.248	0.380		

Note: ***p < 0.001.

Table 6 presents the results of the mediation analyses. First, the total association between SC and MH was 0.579, with a 95% CI [0.517, 0.642], and the direct association was 0.299, with a 95% CI [0.227, 0.370]. This suggests that SC was directly associated with MH and also indirectly associated with MH through resilience. When resilience was included as the mediator, the indirect association was 0.281, with a 95% CI [0.216, 0.345], and the mediated proportion was 48.53% of the total association. This highlights resilience’s significant mediating role in the relationship between SC and MH.

**Table 6:** Mediation analysis of the associations among SC, ACEs, PS, resilience, and MH.

Effect Type	Pathway	Effect Value (*β*)	Percentage (%)	Bootstrap 95% CI		Result
				LLCI	ULCI	
Total Effect	SC → MH	0.579	100%	0.517	0.642	Supported
	ACEs → MH	−0.303	100%	−0.376	−0.230	Supported
	PS → MH	−0.709	100%	−0.764	−0.655	Supported
Direct Effect	SC → MH	0.299	61.64%	0.227	0.370	Supported
	ACEs → MH	−0.157	51.82%	−0.217	−0.098	Supported
	PS → MH	−0.510	71.93%	−0.576	−0.444	Supported
Indirect Effect	SC → Resilience → MH	0.281	48.53%	0.216	0.345	Supported
	ACEs → Resilience → MH	−0.145	47.85%	−0.202	−0.095	Supported
	PS → Resilience → MH	−0.200	28.21%	−0.258	−0.141	Supported

Second, the total association between ACEs and MH was −0.303, with a 95% CI [−0.376, −0.230], and the direct association was −0.157, with a 95% CI [−0.217, −0.098]. This suggests that ACEs were directly associated with MH and also indirectly associated with MH through resilience. With resilience serving as the mediator, the indirect association was −0.145, and the 95% CI ranged from [−0.202, −0.095]. The mediated proportion accounted for 47.85% of the total association, supporting the mediating role of resilience in the association between ACEs and MH.

Finally, the total association between PS and MH was −0.709, with a 95% CI [−0.764, −0.655], and the direct association was −0.510, with a 95% CI [−0.576, −0.444]. This suggests that PS was directly associated with MH and also indirectly associated with MH through resilience. When resilience was included as the mediator, the indirect association was −0.200, with a 95% CI [−0.258, −0.141], and the mediated proportion accounted for 28.21% of the total association, supporting the mediating role of resilience in the association between PS and MH.

## 4. Discussion

This study explored the associations between SC, ACEs, and PS with the MH of college students, with a focus on the mediating role of resilience. Six hypotheses were tested and confirmed. Specifically, resilience was found to strengthen the association between SC and college students’ MH, while also reducing the negative association between ACEs and PS with MH, suggesting potential pathways for improving college students’ MH.

The present findings suggest that demographic characteristics were associated with variation in the study variables. Specifically, older students and those in higher academic grades reported higher levels of SC, resilience, and MH, together with lower PS, which may reflect developmental maturity and a stronger capacity to cope with stress. In addition, only children showed lower PS and more favorable MH profiles than non–only children, which may be related to differences in family support and resource availability. Students whose parents had higher educational levels also tended to report higher SC, resilience, and MH, as well as lower PS, suggesting that family educational background may be linked to more favorable psychological functioning. Although these demographic characteristics were not the primary focus of the present study, they provide important contextual information for interpreting the associations among SC, ACEs, PS, resilience, and MH. Moreover, because these demographic characteristics were controlled as covariates in the mediation analyses, the associations among SC, ACEs, PS, resilience, and MH remained stable after adjustment, indicating that these associations were not substantially explained by demographic characteristics.

The observed association between SC and MH, which reached a moderate-to-large magnitude according to Cohen’s conventions, is consistent with prior findings [25], thus confirming H1. College students who approach personal setbacks with self-understanding tend to interpret challenges less negatively, which aligns with the perspective that compassionate self-attitudes are associated with adaptive emotional regulation. By acknowledging imperfection without self-blame, students may sustain a balanced psychological state even under academic or interpersonal pressure [69]. Practically, integrating reflective or mindfulness-based sessions into student programs may align with more balanced emotional functioning.

The negative association between ACEs and MH, which reached a medium magnitude, is consistent with previous research [15], confirming H2. Specifically, experiences of emotional or physical neglect, abuse, and family conflict during childhood are associated with less adaptive emotional regulation and coping patterns, which may correspond to poorer MH outcomes later in life [35]. These enduring associations underscore the importance of early psychosocial experiences in shaping current emotional responses. Practically, trauma-informed approaches within university counseling services may correspond with improved support for students reporting adverse backgrounds.

The negative association between PS and MH, which reached a large magnitude, is consistent with prior findings [10], confirming H3. This pattern suggests that higher levels of perceived stress are closely linked to poorer psychological functioning among college students. When everyday academic, interpersonal, or future-oriented demands are appraised as exceeding available coping resources, students may experience sustained psychological strain, which is reflected in lower levels of MH [40]. This finding suggests that higher perceived stress may be linked to poorer psychological adjustment among college students. Reducing perceived stress through interventions such as resilience training, emotion regulation workshops, or academic stress management programs may yield noticeable benefits for students’ psychological adjustment and overall well-being.

The findings indicate that resilience is closely involved in the association between SC and MH, with a moderate magnitude, which is consistent with previous research [24], and supports H4. This pattern suggests that students with higher levels of SC tend to report greater resilience, which is concurrently associated with more favorable MH outcomes. Rather than reflecting a direct link alone, the relationship between SC and MH appears to be more comprehensively understood when resilience is considered as an intervening psychological factor. This result extends prior work by highlighting the importance of resilience in explaining how self-compassionate attitudes are associated with psychological well-being among college students [70]. While earlier studies often viewed SC as a direct correlate of MH, the present findings highlight its indirect association through resilience, emphasizing the importance of adaptive coping and emotional flexibility in understanding this relationship. By integrating these constructs, the study contributes to a more comprehensive model of how internal protective factors are linked with students’ MH. Practically, incorporating compassion-focused and resilience-strengthening activities into student programs may align with enhanced psychological adjustment.

Resilience was also found to statistically mediate the association between ACEs and MH in college students, with the indirect association reflecting a small-to-moderate magnitude and accounting for a substantial proportion of the total association. This result is consistent with previous findings [55] and extends prior evidence by demonstrating this mechanism among Chinese college students, confirming H5. Students reporting higher resilience tended to exhibit lower psychological distress even when exposed to early adverse environments, suggesting that resilient functioning may correspond with adaptive emotion regulation and more balanced stress appraisal [54]. This finding expands existing research by indicating that resilience serves not just as a protective factor, but also as a critical psychological resource in managing early adversity and its long-term effects. Unlike earlier studies that primarily focused on the direct negative relationship between ACEs and MH, the present results emphasize the role of resilience in shaping how individuals cope with and integrate past experiences during emerging adulthood. These insights contribute to a more process-oriented understanding of how early stress exposure remains associated with MH through adaptive coping resources. Practically, resilience-enhancement initiatives tailored for students with adverse backgrounds may align with greater emotional balance and psychological stability.

Resilience was also found to be involved in the association between PS and MH in college students, with the indirect association showing a moderate magnitude and accounting for 28.21% of the total association. This result is consistent with previous findings [20] and supports H6. Specifically, students with higher levels of resilience reported fewer negative psychological outcomes under elevated PS, suggesting that resilience may help buffer the impact of stress on well-being. When individuals view PS as manageable rather than overwhelming, they are more likely to employ flexible coping strategies and maintain emotional balance, which is associated with greater psychological stability. This finding extends prior research by demonstrating that resilience not only helps individuals recover from acute adversity but also aids in adapting to persistent, everyday stress [57]. It emphasizes the importance of understanding the relationship between PS and MH through the dynamic regulation of cognitive and emotional resources rather than direct exposure to stress intensity. By identifying resilience as a psychological mechanism linking stress perception and mental well-being, the present study refines existing stress models and emphasizes the role of adaptive flexibility in maintaining stable MH. Practically, integrating resilience training into university stress-management programs may align with improved emotional functioning among students.

### 4.1 Implications

#### 4.1.1 Theoretical implications.

The theoretical contribution of this study lies in several key areas. First, it develops a stress-buffering framework that integrates SC, ACEs, PS, and resilience, revealing their combined impact on college students’ MH. This multidimensional approach extends beyond single-factor analyses, providing a more comprehensive understanding of psychological regulation. Second, by applying the stress-buffering theory, this study highlights resilience as a key mediating mechanism through which SC enhances MH while ACEs and PS undermine coping abilities and increase psychological distress. This reinforces the importance of protective psychological resources in buffering stress effects in academic and life contexts. Third, this study contributes to the literature by positioning resilience as an important explanatory factor in the associations between psychological resources (SC), external stressors (ACEs and PS), and MH. This perspective offers a more nuanced conceptual understanding of stress adaptation and MH in university students. Finally, these findings contribute to the theoretical understanding of how protective psychological resources and stress-related factors are jointly associated with college students’ mental health.

#### 4.1.2 Practical implications.

At the institutional level, the findings may offer tentative implications for university MH education and student support services. Given the observed association between SC and resilience, universities may consider exploring whether compassionate-awareness content could be incorporated into existing MH curricula. For instance, semester-based courses or group counseling modules could include activities such as mindfulness practice, compassion-focused writing, and cognitive reappraisal training as potentially relevant components for supporting students’ emotional adjustment. Student counseling centers might also consider collaborating with faculty mentors to explore peer-support initiatives in which trained peer facilitators guide reflection sessions related to coping and self-kindness. In addition, resilience-related content could be considered in orientation programs, semester workshops, and campus campaigns addressing stress management, emotional literacy, and problem-solving. For students reporting high PS or ACEs, universities may wish to further examine whether trauma-informed counseling, short-term crisis support, or resilience-focused services are appropriate and effective in specific contexts.

At the individual level, the findings indicate that self-reflective and supportive activities may be relevant to students’ mental health. Participation in university-based mental health activities, including small-group counseling, peer dialogue sessions, and online self-compassion courses, may help students develop emotion-regulation skills and stress-coping strategies. In daily life, students may also engage in reflective practices such as guided journaling, meditation, or creative expression through art and music workshops to enhance emotional awareness and self-understanding. Regular engagement in these structured and self-directed activities may support resilience and psychological adjustment in academic and social contexts.

### 4.2 Limitations and future research

While this study highlights the connections between SC, ACEs, PS, resilience, and college students’ MH, and suggests a related mediating model, it is not without limitations. First, the cross-sectional design restricts the ability to infer temporal or causal relationships. Although the results indicate significant associations, it remains uncertain whether changes in SC or resilience precede improvements in MH or vice versa. This limitation constrains the interpretation of directionality and mechanism. Future studies could employ longitudinal tracking or experimental interventions to clarify the developmental and causal pathways among these variables. Second, despite the use of convenience sampling, the coordination of participant recruitment through instructors might have introduced selection bias, as students with higher engagement or interest in psychology may have been more inclined to participate. Independent digital randomization or institution-level stratified sampling could strengthen representativeness in future research. Third, the reliance on self-reported data raises the possibility of social desirability and recall biases, which may have influenced participants’ reporting of stress or emotional states. These biases could affect the accuracy of measured relationships and limit the robustness of conclusions. Future studies could integrate multi-source data—such as teacher ratings, behavioral indicators, or peer assessments—to enhance data validity. Fourth, the operationalization of key constructs in this study was somewhat simplified. Specifically, ACEs were represented using a cumulative total score, and resilience was assessed as an overall construct. Although these approaches are common and analytically practical, they may not fully capture the heterogeneity of different adverse childhood experiences or the potentially multidimensional nature of resilience. Future research could examine specific ACE profiles, distinct forms of adversity, or multiple dimensions of resilience to provide a more nuanced understanding of how these factors are associated with mental health. Finally, the sample consisted primarily of female students (69.7%) from Chinese universities, which may limit generalizability across genders and cultures. Future research could adopt cross-cultural or gender-balanced samples to verify the stability of the proposed model in diverse populations.

## 5. Conclusion

This study examined the relationships among SC, ACEs, PS, resilience, and MH in college students. The findings showed that SC was positively related to resilience and MH, whereas ACEs and PS were negatively related to both. Resilience served as a central mediating factor, highlighting its key role in students’ psychological well-being within a stress-buffering framework. These results suggest that resilience plays a central role in explaining the associations between SC, early adversity, stress, and MH. Practical efforts in higher education could include resilience-building workshops and self-compassion training to promote well-being. Future longitudinal and cross-cultural research is recommended to confirm these associations and further refine intervention strategies.
